# Extraction of Lipids and Functional Properties of Defatted Egg Yolk Powder Obtained Using a One-Step Organic Solvent Lipid Extraction Process

**DOI:** 10.3390/foods13132113

**Published:** 2024-07-02

**Authors:** Casey Showman, Alleda Rose, Cangliang Shen, Jacek Jaczynski, Kristen Matak

**Affiliations:** Division of Animal and Nutritional Sciences, West Virginia University, Morgantown, WV 26505, USAcashen@mail.wvu.edu (C.S.); jacek.jaczynski@mail.wvu.edu (J.J.)

**Keywords:** egg yolk protein, functional properties, organic solvent, lipid extraction

## Abstract

A one-step organic solvent lipid extraction method was used to separate lipids from spray-dried egg yolk. Organic solvents tested were chloroform:methanol (CM, 2:1, *v*:*v*), methyl-tert-butyl ether (MTBE), or hexane:isopropanol (HI, 3:2, *v*:*v*). The resulting defatted egg yolk powder had between 21 and 30% more protein and between 22 and 25% less lipid than the initial spray-dried egg yolk powder (*p* < 0.05). The solubility of the powder decreased from 20% to 4% (*p* < 0.05) when CM was used as the organic solvent, likely due to protein denaturation by the chloroform. Gels made from MBTE and HI-extracted protein concentrates had similar hardness (*p* > 0.05) and were both harder than gels made using the initial egg yolk powder (*p* < 0.05). MTBE gels were springier, more cohesive, and gummier (*p* < 0.05) with similar resistance to the initial egg yolk powder (*p* > 0.05). The results of this study showed that the functionality of the protein in the defatted egg yolk powder was best retained when MTBE was used as the lipid extraction solvent.

## 1. Introduction

Egg yolk contains high-quality nutrients such as protein, fatty acids, vitamins, and minerals. The composition of dried egg yolk is approximately 59% lipid, 34% protein, 1% carbohydrate, and 4% ash [1]. Egg yolk proteins consist of high and low-density lipoproteins with others such as livetins, lipovitellins, and phosvitin. Egg yolk lipid is found exclusively associated with lipoproteins [2]. Phospholipids, specifically phosphatidylcholine from egg yolk, contain omega-3 fatty acids. In addition to its nutritional components, egg yolk exhibits many functional properties, like emulsification and gelation, and is a common ingredient in foods such as mayonnaise and dressings. Low-density lipoproteins are important for gel formation because unfolded proteins form networks using disulfide bonds and hydrophobic interactions. Phospholipids extracted from egg yolk offer many functionalities in the pharmaceutical industry; however, the remaining concentrated protein is often discarded.

Traditional lipid extraction methods such as Folch Bligh and Dyer, and Soxhlet tend to be time-consuming, labor-intensive, and call for the use of chemicals that are not food grade [3,4]; therefore, the effectiveness of one-step strategies are being studied. For example, Rose and colleagues extracted lipids from various insects and krill powder using a 1:10 sample-to-solvent ratio of hexane, hexane:isopropanol (HI, 3:2, *v*:*v*), chloroform, chloroform:methanol (CM, 2:1, *v*:*v*), or methyl-tert-butyl ether (MTBE) as the organic solvents. Samples were simply mixed, centrifuged, and lipids were separated by filtration [5,6,7]. Lipid extraction efficiencies for krill ranged from 9 to 28% [7] and from 23% to 93% in insect flours [5]. As a result of this lipid removal, the defatted powders had up to 80% crude protein recovered from locust flour [6]. This concentrated protein may potentially be used as a food source or functional additive.

Solvents used for lipid extraction range from extremely polar, such as methanol, to non-polar, like petroleum ether, and a combination of polar and non-polar solvents is useful for extracting lipids from egg yolk. Polar solvents denature lipoproteins by disrupting the hydrophilic side chains, exposing the hydrophobic regions, and allowing the non-polar solvents to dissolve the lipids held inside [8]. This structural alteration causes protein aggregation and precipitation, ultimately altering the functionality of the protein. CM is a common solvent combination used in lab settings for lipid extraction, but it is toxic and therefore, not a food-grade solvent. On the other hand, HI is a combination of food-grade solvents that is frequently used as an alternative to CM. Both solvent mixtures employ non-polar and polar components to ensure the removal of neutral and polar lipid classes. Methyl-tert-butyl ether (MTBE) is a chemical produced from methanol and isobutylene, and it is frequently used as an additive in gasoline to replace lead as an octane enhancer. Although not typically used in the food industry, MTBE may be an effective solvent since it is an alkyl ether that has polar tendencies, but is less polar than alcohols; therefore, it will solubilize both polar and non-polar lipids [9]. It should be noted that MTBE is considered a “nuisance chemical” in drinking water; however, there is limited data concerning health risks by human ingestion [10].

It is known that the type of organic solvent used to remove lipids will impact the lipid extraction efficiency; therefore, the first objective of this study was to compare lipid extraction efficiencies and characterize the composition of extracted lipids from egg yolk using a one-step organic solvent extraction process with 2:1 chloroform:methanol (CM), methyl-tert-butyl-ether (MTBE), or 3:2 hexane:isopropanol (HI) as the organic solvents. The impact of the solvents on the proximate composition and functionality of the remaining defatted egg yolk powders has not been studied. Therefore, the second objective of this study was to determine the effects of defatting with organic solvents on the proximate composition and certain functional properties of the defatted egg yolk powders.

## 2. Materials and Methods

Spray-dried egg yolk powder was purchased from Magic Flavors (Seattle, Washington, DC, USA) and stored at −80 °C until analyses were conducted. When powders were defrosted for analysis, they were held at temperatures between 2 and 5 °C. 

### 2.1. Lipid Extraction Process

Lipid removal was performed as described by Rose et al. [6,7]. Briefly, chloroform:methanol (CM; 2:1, *v*:*v*), methyl-tert-butyl ether (MTBE), and hexane:isopropanol (HI; 3:2, *v*:*v*) were used as the extraction solvents (ACS grade chloroform, HPLC grade methanol, HPLC grade MTBE, and ACS grade isopropanol, Fisher Scientific, Fair Lawn, NJ, USA; environmental grade hexane, Alfa Aesar, Ward Hill, MA, USA). First, 3 g of powdered egg yolk was weighed into a 35 mL Teflon-lined Pyrex glass test tube, and 30 mL of the appropriate solvent was added. The sample and solvent mixture were vortexed for 60 s, transferred to a 250 mL beaker with a proportional magnetic stir bar, and mixed on a stir plate for 15 min. The mixture was returned to the test tube and centrifuged at 900*× g* at 10 °C for 10 min. The lipid-containing solvent layer was separated (described below) and the defatted egg yolk powder was dried overnight in a fume hood. The yield of the remaining defatted egg yolk powder remaining after the lipid extraction procedure was calculated after evaporation of the organic solvents using the following formula:

$$\mathrm{Yield}\;(\%)=\frac{weight\;before\;extraction}{redidual\;powder\;weight}\;\times \;100$$

The lipid-containing solvent layer was filtered using a 1-PS filter (Whatman, Buckinghamshire, UK) into a second test tube. Before filtration, the residual silicone was eliminated from the filter paper by rinsing it three times with 5 mL of a 2:1 CM solution. Following filtration, the filter paper was discarded, and both the inside and outside of the funnel were rinsed with a 14.6 cm disposable Pasteur pipette containing a mixture of 2:1 CM. The samples were dried under a nitrogen gas stream in a 60 °C water bath for 60 min to evaporate the residual organic solvents. For thin-layer chromatography, a portion of the lipid layer was transferred to a 14 mL test tube, while the remaining lipid samples were prepared for methylation by adding 125 μL of internal standard (C19; Merck, Darmstadt, Germany). The lipid extraction efficiency was calculated based on the weight of the extracted lipids relative to the concentration of lipids in the initial powder

$$\mathrm{Lipid}\;\mathrm{Extraction}\;\mathrm{Efficiency}\;(\%)=\frac{Lipid\;Extracted\;(g)}{Initial\;Lipid\;(g)}\;\times \;100$$

### 2.2. Fatty Acid Analysis—Lipid Layer Only

#### 2.2.1. Extraction of Fatty Acid Methyl Esters

Fatty acid analysis was conducted on the lipids extracted from each type of organic solvent tested following the method outlined by Rose et al. [5,7]. Methylation was carried out by adding 4 mL of 4% H_2_SO_4_ in anhydrous methanol along with C19, an internal standard used for quantification, and the mixture was held for 60 min in a water bath at 90 °C. Methylation was halted by adding 3 mL of deionized distilled water. Fatty acid methyl esters (FAME) were then extracted using chloroform (8 mL) and filtered through anhydrous Na_2_SO_4_. The resulting mixture was dried under nitrogen gas in a water bath at 60 °C, re-suspended in filtered iso-octane, and stored at −20 °C until analysis.

#### 2.2.2. Gas Chromatography–Flame Ionization Detection

Gas Chromatography–Flame Ionization Detection (GC-FID) analysis was performed using a Varian CP-3800 Gas Chromatograph (Varian Analytical Instruments; Walnut Creek, CA, USA) equipped with a flame ionization detector (FID; Varian Inc., Walnut Creek, CA, USA) and a silica capillary column (100 m length, 0.25 mm diameter) for separation. The temperature protocol commenced at 140 °C for 5 min, followed by a gradual temperature ramp of 4 °C per min until reaching 220 °C, where it was maintained for 15 min. The entire separation process for FAME lasted 85 min. Injector and detector temperatures were maintained at 270 °C and 300 °C, respectively. Identification of FAME was achieved by comparing retention times to those of the FAME 37 standard (Merck, Darmstadt, Germany). Peak areas and the relative quantities of each fatty acid in the samples were determined using the Star GC workstation (version 6, Varian Analytical Instruments, Walnut Creek, CA, USA).

### 2.3. Thin Layer Chromatography—Lipid Layer Only

Thin layer chromatography (TLC) was used to identify the lipid classes extracted from egg yolk powders, following the method outlined by Rose et al. [5,7]. Extracted lipids were diluted with a 1:1 chloroform–methanol solvent (1 mL) and standards representing the various lipid classes and a portion of each sample were transferred to a 20 cm × 20 cm silica gel plate (Merck TLC silica gel 60 W F_254 s plates with 60 A pore size, Darmstadt, Germany). During the mobile phase, the plates were placed in a glass chamber with hexane–diethyl ether–acetic acid (80:20:1; *v*:*v*:*v*). After 1 h, 50% sulfuric acid was sprayed onto the plates, and they were dried overnight. Additional drying was performed in a drying oven at 110 °C for 40 min. Pixel density was measured using the GelDocing system (Bio-Rad Gel Doc XR+ and ChemiDoc XRS+ Imaging Systems with Image Lab Software Version 6, Hercules, CA, USA).

### 2.4. Proximate Composition

Samples of defatted egg yolk powder from each solvent extraction (CM, MTBE, and HI) and initial egg yolk powder were analyzed for proximate composition (moisture, ash, crude protein, and lipid). All procedures followed AOAC-approved methods [11]. Briefly, the moisture content of the samples was determined using the oven-drying method. Samples were placed in a 100 °C oven overnight. The following formula was used to determine moisture content [12]:

$$\mathrm{Moisture}\;\mathrm{content}\;(\mathrm{MC})=\frac{initial\;weight\;-\;oven\;dried\;weight}{initial\;weight}\;\times \;100\;\mathrm{of total mass}$$

Samples that had been previously dried for the moisture content analysis were then incinerated in an A1500 furnace (F-A1525M-1; Thermolyne Corporation; Dubuque, IA, USA) muffle oven overnight at 550 °C. The ash content was determined by the following formula [12]:

$$\mathrm{Ash}\;\mathrm{content}\;(\mathrm{AC})=\frac{dried\;weight}{inital\;weight}\;\times \;100\;\mathrm{of total mass}$$

Crude protein was measured by Kjeldahl N assay using the Kjeltec 2300 (Foss North America; Eden Prairie, MN, USA). The steps of the assay included digestion, distillation, and titration. Samples (0.2 g) and Kjel-tabs were dissolved in 10 mL of sulfuric acid. Samples were digested by heating at 400 °C for 50 min with constant vacuum by an exhaust manifold. After digestion, 25 mL of distilled water was added, and the solution was titrated. The amount of acid (mL) required to titrate the solution was used to determine the nitrogen content in moles. The moles of nitrogen were multiplied by 14.01 (N atomic number) to obtain grams of nitrogen, and thereby crude protein was calculated as grams of nitrogen by 6.25.

$$\mathrm{Crude}\;\mathrm{protein}\;(\mathrm{CP})=\frac{0.1\;N\;HCl\;\times \;14.01\;\times \;6.25\;\times \;mLs\;acid}{sample\;weight\;\times \;1000}\;\times \;100\;\mathrm{of total mass}$$

Crude lipid content was measured by Soxhlet extraction with petroleum ether where 0.5 g of sample was folded in Whatman 41 (Whatman, 1441-125) filter paper. Following 18 h extraction, samples were dried overnight at 100 °C. Fat content, on a dry matter basis, was determined with the following formula [11]:

$$\mathrm{Crude}\;\mathrm{lipid}\;\mathrm{content}=\frac{\left(inital\;weight\;-\;dried\;weight\right)-grams\;moisture}{initial\;weight}\;\times \;100\;\mathrm{of total mass}$$

### 2.5. Protein Solubility

Initial egg yolk powder and defatted powders (CM, MTBE, and HI) were analyzed for water-soluble protein content. Firstly, 2 g of each sample was reconstituted with 20 mL of distilled water. The mixture was stirred for 30 min at room temperature and transferred to a 35 mL test tube. The tubes were centrifuged at room temperature for 30 min at 5000× *g* to separate water-soluble and water-insoluble proteins. The supernatant was decanted into freeze-dry cups, and both the water-soluble and water-insoluble fractions were freeze-dried (VirTis Freeze Dryer, SP Scientific, Stone Ridge, NY, USA). The protein content of each was calculated by the Kjeldahl assay previously mentioned. Protein solubility was calculated using the following formula and reported on a dry matter basis:

$$\%\;\mathrm{soluble}\;\mathrm{protein}=\frac{protein\;content\;of\;water\;-\;soluble\;fraction\;(g)}{initial\;protein\;in\;sample\;(g)}\;\times \;100$$

### 2.6. Gelling Properties

The gelling properties of the initial egg yolk powder and defatted powders (CM, MTBE, and HI) were measured by reconstituting the powders to a moisture content of 80% (*w*/*w*) with 0.5 M NaCl salt solution in distilled water. The salt solution was used to increase solubility and denature proteins to form a gel [13]. The reconstituted powders were poured into lightly oiled 50 mL plastic tubes and capped. Samples were cooked in a 90 °C water bath for 20 min, and then immediately cooled on ice to room temperature. Gels were stored in tubes overnight at 4 °C. Six tubes of each gel type were prepared for analysis.

### 2.7. Texture Profile Analysis of Gels

Previously cooked gels were removed from tubes and cut into cylinders measuring 2.54 cm in both height and diameter. Texture profile analysis (TPA) of the prepared gels was conducted using a 70 mm diameter plate probe mounted to the TA-HDi Texture Analyzer (Texture Technologies Corp.; Scarsdale, NY, USA). Samples were subjected to a double compression test (25% compression) with a 50 kg load cell and a speed of 1 mm/s. The maximum force (g) observed during compression was used to analyze hardness, springiness, cohesiveness, gumminess, and resilience. TPA was analyzed by the Texture Expert Exceed software (version 2.60; Stable Micro Systems Ltd., Surrey, UK). At least six gel samples were tested per solvent type.

### 2.8. Water-Holding Capacity of Gels

The water-holding capacity (WHC) of gels measuring 2.54 cm in both height and diameter was analyzed by a uniaxial compression test using a 70 mm diameter plate probe mounted to the TA-HDi Texture Analyzer (Texture Technologies Corp.; Scarsdale, NY, USA) with a 50 kg load cell and a speed of 1 mm/s. Gels were placed between two dry Whatman G08 filter papers and double compressed to 50%. Water-holding capacity was determined by the following equation:

$$\mathrm{WHC}\;(\%)=\frac{initial\;water\;content\left(g\right)-water\;lost\;(g)}{inital\;water\;content\;(g)}\;\times \;100$$

The initial water content was determined by moisture analysis with the previously mentioned methods. Water loss was determined by weighing the filter paper before and after compressions and taking the difference. At least six gel samples were tested per solvent type.

### 2.9. Color Analysis of Gels

Color analysis of gels from each sample type were measured using a colorimeter (Minolta Camera Co., Ltd., Osaka, Japan) calibrated with a standard white plate. Whiteness was calculated after measuring L* (lightness: 0–100), a* (intensity of red color: −60 to +60), and b* (intensity of yellow color: −60 to +60).

Whiteness = 100 − [(100 − *L*)^2^ + *a*^2^ + *b*^2^]^1/2^

At least six color measurements of gels were taken per solvent type.

### 2.10. Statistical Analysis

Lipid extractions were replicated three times for each solvent used in the methodology. For each triplicate, fatty acid analyses were conducted in triplicate and TLC was duplicated. One-way analysis of variance (ANOVA) was used to determine variations between treatments and Student’s *t*-test was used to compare means. Defatted egg yolk powders from each solvent extraction were mixed to make a bulk homogenate sample for each CM, MTBE, and HI. Proximate composition was conducted to verify composition. The proximate composition of the protein concentrates was completed in duplicates for each of the extraction replicates and compared to the initial egg yolk powder. The functional properties of water solubility and gelling (texture profile analysis, water-holding capacity, and color) were all replicated at least three times and compared to the initial egg yolk powder. The results are reported as mean ± standard deviation (SD). Statistical analyses were conducted using SAS JMP version 13 (SAS Institute Inc., Cary, NC, USA). One-way independent measures analysis of variance (ANOVA) was used to determine individual differences between treatments. Post hoc analysis was conducted using Student’s *t*-test with a significance level of (*p* < 0.05).

## 3. Results and Discussion

### 3.1. Lipid Extraction Efficiency, Fatty Acid Profile, and Classes of Extracted Lipids

Lipid extraction efficiencies from the egg yolk powder averaged around 30% (Table 1), which is consistent with the results reported by Rose and others when they used CM as the organic solvent to remove lipids from krill [7]. They also reported that when lipids were removed from both krill and insect powders, lipid extraction efficiencies were best when CM was used as the organic solvent [5,7]. In this current study, lipid extraction by CM was numerically greater, yet not significantly different from the other solvents tested (*p* > 0.05). In addition, the relative fatty acid composition of lipids separated from egg yolk powder was consistent, regardless of the organic solvent used (Table 1).

**Figure 1:**
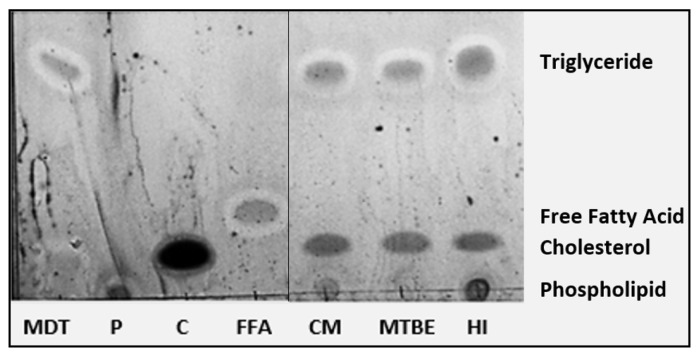
Separated lipid classes of egg yolk powders following a one-step organic solvent extraction with various organic solvents. Standards: MDT = monoglyceride, diglyceride, triglyceride; P = phospholipid; C = cholesterol; FFA = free fatty acid. Organic Solvents: CM = chloroform:methanol; MTBE = methyl-tert-butyl-ether; HI = hexane:isopropanol.

Figure 1 shows the clear separation of the lipid classes recovered using the one-step lipid extraction by thin layer chromatography (TLC), and densitometry analysis was used to quantify these classes (Table 1). Egg lipids are concentrated in the yolk (up to 60%) and consist mainly of triglycerides (62% dry basis) which will readily dissolve in non-polar or low-polar organic solvents like hexane or chloroform, respectively. The proportion of triglycerides extracted from the egg yolk powder was consistent with reported values of triglycerides ranging from 54 to 64% with the greatest when HI was used as the extraction solvent (*p* < 0.05).

Egg yolk contains a significant concentration of phospholipids (33% on a dry basis) [2] and the proportion of phospholipids was lower than reported values, ranging from 16 to 26% (Table 1). More polar solvents are better suited than low-polar solvents for dissolving these types of lipids. Polar solvents are characterized by a large dipole moment and contain bonds between atoms with distinct electronegativities, like oxygen and hydrogen in a water molecule. Methanol and isopropanol belong to the class of alcohol organic compounds and are examples of polar solvents; however, they are miscible in water, and so when they are used in a lipid extraction process, their main function is not to dissolve lipids but to disrupt the electrostatic forces between lipids and proteins [14]. Therefore, alcohols are often paired with non- or low-polar solvents in lipid separation strategies.

Egg yolk also contains cholesterol (5% dry basis) [2] and densitometry showed the proportion of cholesterol in the separated lipid to be much higher than reported values, ranging from 20 to 28% (Table 1). Cholesterol is non-polar yet has been shown to dissolve in polar alcoholic organic solvents; therefore, it is likely that some of it was dissolved by the methanol and isopropanol. It is not surprising that when MTBE was used as the extraction solvent, the separated lipid had the greatest proportion of cholesterol (*p* < 0.05) as MTBE was historically used in human medicine to dissolve cholesterol gallstones [15].

### 3.2. Yield and Proximate Composition of Defatted Egg Yolk

The yield of defatted egg yolk after lipid extraction ranged from 46 to 57% with statistically lesser yields when CM was used as the organic solvent (Table 2; *p* < 0.05). This is likely a result of the numerically greater lipid extraction efficiency of CM. The initial powder contained 49% lipid and 36% protein (Table 2). The proximate composition of the defatted egg yolk powders showed that the organic solvents tested significantly reduced lipid composition to less than 28% lipid when HI was used as the organic solvent, 26% lipid when CM was used, and the greatest reduction of lipid (24%) occurred when MTBE was used as the organic solvent (*p* < 0.05). Protein was concentrated to more than 57% in the defatted samples (Table 2; *p* < 0.05). Even though it had the lowest yield, when CM was used as the organic solvent, samples had the greatest concentration of protein (66%; *p* < 0.05), while egg yolk powders defatted using HI and MTBE showed similar protein concentrations (57 to 58%; *p* > 0.05).

### 3.3. Protein Solubility

**Figure 2:**
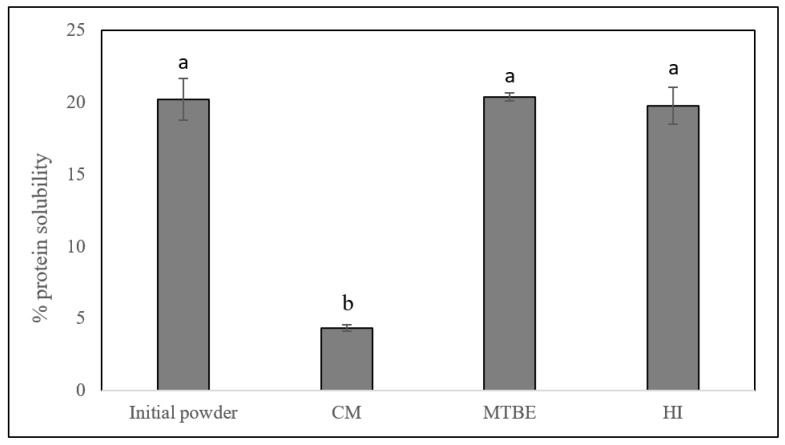
Protein solubility (%) of initial egg yolk powder and defatted powders separated using 2:1 chloroform:methanol (CM), methyl-tert-butyl-ether (MTBE), or 3:2 hexane:isopropanol (HI) as the organic solvent. ^a,b^ Different letters indicate significant differences (*p* < 0.05) among organic solvents.

The solubility of the protein in the defatted egg yolk and initial powder was measured, and the results are expressed as percent soluble protein (Figure 2). The protein in the initial egg yolk powder had a solubility of 20% and the solubility of the protein in the egg yolk defatted using HI and MTBE was not altered (*p* > 0.05). This is consistent with Rose and others (2023) who measured the solubility of protein concentrates extracted from cricket or locust powders and reported that when MTBE was used as the extraction solvent, there were no significant differences in protein solubility on a pH gradient from pH 5.0 to pH 11.0 [6]. On the other hand, in this current study when CM was used as the extraction solvent for egg yolk lipids, the solubility of the protein in the defatted egg yolk was significantly reduced from 20% to 4% (*p* < 0.05) which is consistent with other studies. For example, Rezig and colleagues reported that the protein in pumpkin seed flour defatted using 3:1 chloroform–methanol (flour–solvent: 1:10 *w*:*v*) was about 55% soluble, whereas the protein in pumpkin seed flour defatted using pentane had a solubility that reached 80% [16]. The reduction in solubility is likely due to the chloroform, which is a low-polar chlorinated hydrocarbon and is often used as a protein-denaturing agent. In addition, the polar solvent methanol interrupts hydrogen bonding and electrostatic interactions in the protein which could in turn alter the equilibrium of the hydrophilic and hydrophobic interactions responsible for water solubility [8].

### 3.4. Protein Gel Formation and Texture Profile Analysis

The ability of proteins to form gels is strongly dependent on changes in the protein structure and aggregation interactions; therefore, gelation and the texture profiles of the gels made from the initial egg yolk powder and the defatted egg yolk powders were compared. The results showed that the gelling abilities and texture profiles were impacted by the type of organic solvent used. For example, CM and other organic solvents are known to cause denaturation to proteins and to reduce their functional properties; accordingly, when CM was used as the organic solvent, the resulting protein concentrates did not form a solid gel.

Texture profiles (hardness, springiness, gumminess, cohesiveness, and resilience) of the gels made from initial egg yolk and the egg yolk powders defatted using HI and MTBE were evaluated (Table 3). The hardness of a gel is measured by the peak force of the first compression. MTBE and HI gels were significantly harder (*p* < 0.05) than the gels prepared with the initial egg yolk powder likely because they contained almost twice the concentration of protein than the initial powder. The cohesiveness of a gel is related to how well it holds together under two compression cycles, and MTBE gels exhibited significantly greater cohesiveness than HI and initial powder gels (*p* < 0.05). MTBE gels were also springier and gummier (*p* < 0.05) which relates back to the hardness of the gel. These results indicate that the MTBE gels would require more mastication energy to physically breakdown the product.

### 3.5. Water-Holding Capacity

**Figure 3:**
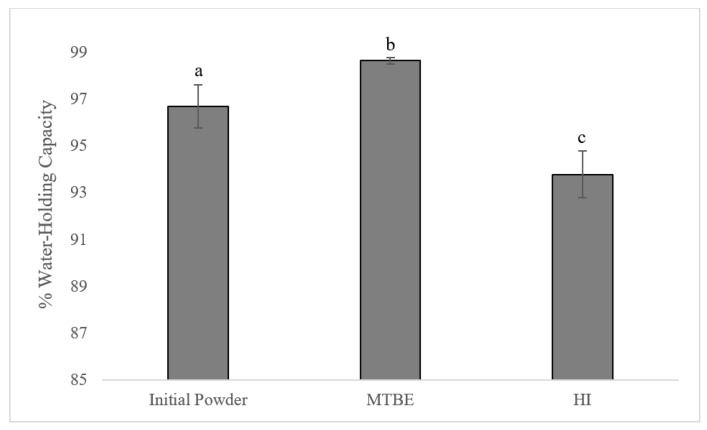
Water-holding capacity (%) of gels prepared with initial egg yolk powder and defatted powders separated using methyl-tert-butyl-ether (MTBE) or 3:2 hexane:isopropanol (HI) as the organic solvent. ^a,b,c^ Different letters indicate significant differences (*p* < 0.05) among organic solvents.

Water-holding capacity (WHC) is a measurement of the water-binding ability of the protein gels and impacts many characteristics of a product including physical, chemical, and sensory attributes. WHC was measured by calculating the weight of water lost during compression and the results are expressed as percent (%) water held in the gel (Figure 3). With over 98%, MTBE gels had the greatest water-holding capacity (*p* < 0.05). Even though HI gels had greater values for TPA than gels from the initial egg yolk, at 93%, the WHC was significantly lower (*p* < 0.05).

### 3.6. Color

The color of a food product is an important characteristic for consumer acceptance. Color measurements (L*, a*, b*) of the gels prepared with the defatted egg yolk powders and the initial egg yolk powder were compared (Table 3). Even though the egg yolk powder defatted using 2:1 CM did not produce a gel, color measurements were still taken for comparison. Gels made from the initial egg yolk powder were the lightest (*p* < 0.05) followed by gels prepared from HI and MTBE defatted egg yolk, which had similar L* values (*p* > 0.05). The gels made from the initial egg yolk powder were also significantly more yellow than the other samples (*p* < 0.05). The color of egg yolk is attributed to the fat-soluble pigments such as carotenoid-like xanthophylls found within the yolk [2]. The xanthophylls are obtained from corn or additives in food fed to poultry. β-carotene is a precursor to the lipid-soluble vitamin A and also contributes to the color of the yolk [8]. Therefore, removing a portion of the lipid content also removed the color attributes of the defatted powder. Kovalcuks and Duma found that lipid extraction employing isopropanol–hexane extracted more β-carotene than the ethanol–chloroform mixture [8]. The CM gels in this study had less yellow color than the HI gels, but this could have been due to the CM gel having a redder color as well as being tested as a liquid rather than a gel like the HI sample.

## 4. Conclusions

The results of this study indicate that one-step organic solvent lipid extraction using chloroform:methanol (CM) (2:1, *v*:*v*), methyl-tert-butyl ether (MTBE), or hexane:isopropanol (HI) (3:2, *v*:*v*) influences the functional properties of defatted egg yolk powders. Utilizing different solvent combinations reduced the lipid content of the egg yolk powder while increasing the protein content. When CM and MTBE were used as the organic solvent, the defatted egg yolk powders had similar concentrations of remaining lipid. The egg yolk powders defatted using CM had the greatest protein content; however, the functional properties were significantly altered. When MTBE was used to defat egg yolk powders, the functional properties were most like the initial egg yolk powder. The MTBE-defatted powders also showed no change in water solubility, produced a stronger, harder gel, and had greater water-holding capacity when compared to the other samples. A potential use for these defatted powders in the food industry is as an ingredient to enhance the nutritional quality or functional properties of an otherwise low-protein food item; however, it is important to note that solvent residues in the defatted egg yolk powders were not measured, and it is possible that levels could exceed the allowable levels in food products. Therefore, solvent removal should be optimized, and residues measured before commercialization.

## Figures and Tables

**Table 1:** Lipid extraction efficiencies (% of initial powder), fatty acid profile (% sample), thin layer chromatography (TLC), and densitometry analysis (%) of lipids following one-step organic solvent extraction using 2:1 chloroform:methanol (CM), methyl-tert-butyl-ether (MTBE), or 3:2 hexane:isopropanol (HI) as the organic solvent.

	CM	MTBE	HI
Lipid Extraction Efficiency	31.96 ± 1.70	30.42 ± 4.16	28.13 ± 4.81
Fatty Acids			
C14:0	0.16 ± 0.01	0.19 ± 0.01	0.17 ± 0.00
C16:0	40.96 ± 1.48	39.41 ± 1.54	40.70 ± 0.08
C16:1n9	0.00 ± 0.00	0.03 ± 0.04	0.00 ± 0.00
C17:1n10	0.11 ± 0.00	0.11 ± 0.00	0.10 ± 0.00
C18:0	0.11 ± 0.00	0.12 ± 0.00	0.11 ± 0.00
C18:1n9t	2.36 ± 0.11	2.16 ± 0.24	2.23 ± 0.01
C18:1n9	41.06 ± 1.67 ^b^	43.54 ± 0.56 ^a^	39.74 ± 0.25 ^b^
C18:2n6	0.02 ± 0.00	0.01 ± 0.01	0.01 ± 0.00
C18:3n3	0.05 ± 0.07 ^b^	0.33 ± 0.12 ^a^	0.34 ± 0.01 ^a^
C20:0	14.46 ± 0.06	14.00 ± 0.11	14.75 ± 0.16
C20:2n6	0.01 ± 0.00	0.02 ± 0.02	0.02 ± 0.02
C20:4n6	1.31 ± 0.10	1.00 ± 0.41	1.26 ± 0.04
C20:4n3	0.04 ± 0.01	0.02 ± 0.00	0.03 ± 0.00
C20:5n3 (EPA)	0.00 ± 0.00	0.00 ± 0.00	0.00 ± 0.0
C22:0	0.45 ± 0.12	0.35 ± 0.15	0.10 ± 0.03
C22:1n9	0.12 ± 0.00	0.10 ± 0.02	0.05 ± 0.05
C22:6n3 (DHA)	0.39 ± 0.03	0.29 ± 0.14	0.38 ± 0.01
Lipid Classes (TLC)			
Triglycerides	53.84 ± 3.89 ^b^	56.03 ± 5.39 ^b^	63.87 ± 4.97 ^a^
Phospholipids	25.54 ± 2.83 ^a^	15.72 ± 3.36 ^b^	16.32 ± 1.34 ^b^
Cholesterol	20.63 ± 1.08 ^b^	28.26 ± 2.97 ^a^	19.80 ± 3.64 ^b^
Free Fatty Acids	0.00 ± 0.00	0.00 ± 0.00	0.00 ± 0.00

^a,b^ Different letters indicate significant differences (Student’s *t*-test, *p* < 0.05) between mean values (±SD, *n* = 3) within the same row.

**Table 2:** Yield (%) of defatted egg yolk powder after lipid extraction and proximate composition (% crude protein, crude lipid, ash, moisture) of initial egg yolk powder and defatted powders separated using 2:1 chloroform:methanol (CM), methyl-tert-butyl-ether (MTBE), or 3:2 hexane:isopropanol (HI) as the organic solvent.

	Initial Powder	CM	MTBE	HI
Yield	N.A.	46.55 ± 0.51 ^b^	55.61 ± 1.77 ^a^	57.12 ± 1.68 ^a^
Crude Protein	36.09 ± 0.16 ^c^	66.19 ± 0.55 ^a^	57.93 ± 0.39 ^b^	57.27 ± 0.63 ^b^
Crude Lipid	49.47 ± 0.93 ^a^	25.67 ± 0.40 ^c^	24.29 ± 0.77 ^c^	27.70 ± 0.64 ^b^
Ash	6.31 ± 0.04 ^c^	9.17 ± 0.41 ^ab^	8.71 ± 0.17 ^b^	9.38 ± 0.12 ^a^
Moisture	3.15 ± 0.12 ^c^	5.15 ± 0.19 ^ab^	5.44 ± 0.10 ^a^	4.80 ± 0.27 ^b^

N.A., not applicable. ^a,b,c^ Different letters indicate significant differences (Student’s *t*-test, *p* < 0.05) between mean values (±SD, *n* = 3) within the same row.

**Table 3:** Texture profile analysis and color measurements of gels prepared with initial egg yolk powder and defatted powders separated using 2:1 chloroform:methanol (CM), methyl-tert-butyl-ether (MTBE), or 3:2 hexane:isopropanol (HI) as the organic solvent.

	Initial Powder	CM	MTBE	HI
Hardness (g)	183.65 ± 10.34 ^b^	n.d.	654.74 ± 75.41 ^a^	642.70 ± 81.95 ^a^
Springiness (mm)	0.59 ± 0.02 ^c^	n.d.	0.83 ± 0.03 ^a^	0.73 ± 0.03 ^b^
Cohesiveness (ratio)	0.57 ± 0.01 ^c^	n.d.	0.66 ± 0.01 ^a^	0.60 ± 0.01 ^b^
Gumminess (g)	97.77 ± 7.89 ^c^	n.d.	406.32 ± 43.94 ^a^	359.55 ± 52.43 ^b^
Resilience (ratio)	0.50 ± 0.01 ^a^	n.d.	0.48 ± 0.01 ^a^	0.39 ± 0.01 ^b^
L*	90.98 ± 0.75 ^a^	77.25 ± 1.40 ^c^	84.62 ± 1.33 ^b^	86.06 ± 0.92 ^b^
a*	−4.89 ± 0.34 ^a^	0.39 ± 0.25 ^a^	−5.77 ± 0.17 ^c^	−4.54 ± 0.30 ^b^
b*	39.89 ± 1.42 ^a^	29.45 ± 0.83 ^c^	30.75 ± 0.47 ^c^	32.66 ± 1.24 ^b^
Whiteness	58.80 ± 1.35 ^c^	62.77 ± 1.15 ^b^	65.12 ± 0.80 ^a^	64.19 ± 1.00 ^ab^

^a,b,c^ indicates significant differences (*p* < 0.05) in rows among the different egg yolk samples. n.d. indicates samples did not form a gel.

## Data Availability

The original contributions presented in the study are included in the article, further inquiries can be directed to the corresponding author.
